# International Spread of Multidrug-Resistant *Campylobacter coli* in Men Who Have Sex With Men in Washington State and Québec, 2015–2018

**DOI:** 10.1093/cid/ciz1060

**Published:** 2019-10-29

**Authors:** Alexander L Greninger, Amin Addetia, Kimberly Starr, Robert J Cybulski, Mary K Stewart, Stephen J Salipante, Andrew B Bryan, Brad Cookson, Christiane Gaudreau, Sadjia Bekal, Ferric C Fang

**Affiliations:** 1 Departments of Laboratory Medicine and Microbiology, University of Washington School of Medicine, Seattle, Washington, USA; 2 Department of Pathology and Area Laboratory Services, Brooke Army Medical Center, San Antonio, Texas, USA; 3 Microbiologie médicale et infectiologie, Centre hospitalier de l’Université de Montréal, Québec, Canada; 4 Département de microbiologie, infectiologie et immunologie, Université de Montréal, Québec, Canada; 5 Laboratoire de santé publique du Québec, Institut national de santé publique du Québec, Sainte-Anne-de-Bellevue, Québec, Canada

**Keywords:** *Campylobacter*, MSM, antimicrobial resistance, Washington State, Québec

## Abstract

**Background:**

*Campylobacter* species are among the most common causes of enteric bacterial infections worldwide. Men who have sex with men (MSM) are at increased risk for sexually transmitted enteric infections, including globally distributed strains of multidrug-resistant *Shigella* species.

**Methods:**

This was a retrospective study of MSM-associated *Campylobacter* in Seattle, Washington and Montréal, Québec with phenotypic antimicrobial resistance profiles and whole genome sequencing (WGS).

**Results:**

We report the isolation of 2 clonal lineages of multidrug-resistant *Campylobacter coli* from MSM in Seattle and Montréal. WGS revealed nearly identical strains obtained from the 2 regions over a 4-year period. Comparison with the National Center for Biotechnology Information’s Pathogen Detection database revealed extensive *Campylobacter* species clusters carrying multiple drug resistance genes that segregated with these isolates. Examination of the genetic basis of antimicrobial resistance revealed multiple macrolide resistance determinants including a novel ribosomal RNA methyltransferase situated in a CRISPR (clustered regularly interspaced short palindromic repeats) array locus in a *C. coli* isolate.

**Conclusions:**

As previously reported for *Shigella,* specific multidrug-resistant strains of *Campylobacter* are circulating by sexual transmission in MSM populations across diverse geographic locations, suggesting a need to incorporate sexual behavior in the investigation of clusters of foodborne pathogens revealed by WGS data.

## Teaser

We show defined clonal lineages of likely sexually transmitted *Campylobacter* species associated with spread in men who have sex with men between Seattle and Montréal. We also report the first CRISPR-acquired antibiotic resistance gene in a clinical isolate.

Men who have sex with men (MSM) are at increased risk for sexually transmitted enteric infections including shigellosis, campylobacteriosis, giardiasis, and viral hepatitis [1]. Sexually transmitted enteric pathogens in MSM are becoming increasingly resistant to first-line antimicrobial agents. Global transmission of azithromycin-resistant *Shigella flexneri* serotype 3a associated with MSM has been shown across the United Kingdom, United States, Canada, and Australia, and possibly Latin America, during the past 20 years [2]. Outbreaks of *Shigella sonnei* resistant to ciprofloxacin and/or azithromycin associated with MSM have also been detected over the past decade in the United States, Canada, Taiwan, and Australia [3–6].

*Campylobacter* species (spp) are common causes of bacterial gastroenteritis worldwide that have been recognized as a cause of sexually transmitted infections in MSM since before the onset of the human immunodeficiency virus (HIV) epidemic [7–9]. Although gradually increasing antimicrobial resistance in clinical *Campylobacter* isolates has been noted [10], a specific association between multidrug-resistant *Campylobacter* and MSM has been reported only sporadically. Fluoroquinolone resistance was common among *Campylobacter* isolates from people living with HIV in France from 1989 to 1994, but macrolide resistance was infrequent [11]. During the past 2 decades, several outbreaks of multidrug-resistant *Campylobacter* have occurred in MSM in Québec, Canada. The first occurred in 1998–2001, involving 11 men with enterocolitis caused by erythromycin- and ciprofloxacin-resistant *Campylobacter jejuni* that was clonal by pulsed-field gel electrophoresis [12]. Two discrete clades of macrolide- and fluoroquinolone-resistant *C. jejuni* were noted to be persistent in the MSM community in Québec between 2003 and 2013 [13]. From 2010 to 2011, tetracycline- and ciprofloxacin-resistant *Campylobacter coli* pulsovar 1 was detected among 10 MSM in Montréal, while a *C. coli* pulsovar 15 outbreak involved 6 MSM in Montréal in 2015 [14, 15]. Most recently, a cluster of *Campylobacter fetus* infections occurred in Québec in 2014–2016, including several fluoroquinolone-resistant strains [16].

Over the past 3 years, we noted several multidrug-resistant *Campylobacter* isolates from patients in the Seattle, Washington area, some of whom were MSM. Here, we present the epidemiological correlates, antimicrobial resistance phenotypes, and genotypic characterization based on whole genome sequencing (WGS) of these isolates along with comparisons to 9 multidrug-resistant *C. coli* isolates obtained from MSM in Québec during 2015–2018.

## METHODS

### Isolate Description and Antimicrobial Susceptibility

This study was approved by the University of Washington Institutional Review Board and the Centre hospitalier de l’Université de Montréal Research Ethics Committee. For Washington State isolates, stools that were positive on the FilmArray Gastrointestinal Panel (BioFire Diagnostics, Salt Lake City, Utah) for *Campylobacter* spp were plated on *Campylobacter* CVA selective media and incubated for 3 days at 42°C in microaerophilic conditions. Following incubation, *Campylobacter* spp were identified by a matrix-assisted laser desorption/ionization–time of flight (MALDI-TOF) mass spectrometry system (Bruker Daltonics, Bremen, Germany).

All *Campylobacter* isolates were grown for 24 hours at 37°C in microaerophilic conditions before susceptibility testing. *Campylobacter jejuni* ATCC 33560 was used as a quality control. After 24 hours, several colonies were suspended in saline to a turbidity of 0.5 McFarland. Isolates were cultured on Mueller-Hinton agar with 5% sheep blood agar under microaerophilic conditions at 37°C for 24 hours. Etests (bioMérieux, Marcy-l’Étoile, France) containing ciprofloxacin, amoxicillin–clavulanic acid, erythromycin, fosfomycin, gentamicin, meropenem, tetracycline, rifampin, azithromycin, clindamycin, and chloramphenicol were used to determine minimum inhibitory concentrations (MICs). A portion of the antimicrobial susceptibility testing (AST) was performed retrospectively for nonclinical purposes. Dual macrolide- and fluoroquinolone-resistant isolates were chosen for WGS.

### *Campylobacter* Sequencing and Analysis

DNA was extracted from *Campylobacter* pellets using the MoBio UltraPure kit. DNA was quantitated on a Qubit 3.0 fluorometer (Thermo Fisher) and 1 ng was used as input for tagmentation library preparation using two-fifths volumes of the Nextera XT kit protocol (Illumina). Libraries were amplified using dual-index primers with 17 cycles of polymerase chain reaction and purified using 0.7X Ampure beads. Libraries were quantitated using the Qubit 3.0 fluorometer and sequenced on a 2 × 300 bp run of the Illumina MiSeq. Sequencing reads and assembly contigs are available in the National Center for Biotechnology Information (NCBI) under BioProject PRJNA542889.

Additional *Campylobacter* isolates that fell into single-nucleotide polymorphism (SNP) clusters (based on single-linkage clusters with a maximum of 50 SNP differences) with the 18 sequenced isolates were identified using the NCBI Pathogen Detection browser (as of 15 June 2019) and included in downstream bioinformatic analyses. BioSample metadata were also downloaded for 8624 *Campylobacter* isolates in Pathogen Detection that were collected between June 2014 and January 2019 and derived from clinical material. Of these, 5039 isolates contained age ranges. Age ranges were set in 20-year bins and distribution between sequenced clinical *Campylobacter* isolates available in NCBI, and isolates clustering with our sequenced isolates were compared using Fisher exact test.

Paired-end sequencing reads were adapter- and quality-trimmed using trimmomatic (ILLUMINACLIP:NexteraPE-PE.fa:2:30:10 LEADING:3 TRAILING:3 SLIDINGWINDOW:4:30 MINLEN:20) and de novo assembled using SPAdes version 3.13 with default options followed by prokka version 1.13 annotation of contigs [17, 18]. Antibiotic resistance genes were identified using the NCBI AMRFinder [19]. Virulence genes were identified using VFanalyzer and a custom BLAST database constructed with corresponding genes from the *C. jejuni* NCTC11168 strain (NC_002163.1) as well as *virB11* (NC_005012.1) and *hcp* (NZ_CP028373.1) [20]. The presence and absence of virulence genes are discussed in the Supplementary Materials. Multilocus sequence typing (MLST) was performed using PubMLST and Torsten Seeman’s MLST typing tool (https://github.com/tseemann/mlst) [21]. Initially unassigned sequence types and alleles were submitted to PubMLST to obtain definitions.

Core genome SNP phylogeny was performed as described previously and used to initially discover isolate clusters and confirm NCBI Pathogen Detection relationships [6, 22]. In brief, quality- and adapter-trimmed reads were aligned to a reference genome with BWA-MEM and SAMtools and filtered with VCFtools using the following parameters: minDP 10, minQ 200, and minGQ 10. The clinical *C. jejuni* NCTC11168 strain (NC_002163.1) was used as the reference genome for constructing the *C. jejuni* phylogeny [23]. The multidrug-resistant *C. coli* RM2228 strain (NZ_CP035927.1) was used as the reference genome for constructing *C. coli* phylogeny [24].

## RESULTS

### Bacterial Strains

Nine isolates of *Campylobacter* spp resistant to both fluoroquinolones and macrolides were isolated by the clinical microbiology laboratories at Harborview Medical Center (Seattle, Washington) or the University of Washington Medical Center between 2015 and 2018. Three were *C. jejuni* and 6 were *C. coli*, as identified by MALDI-TOF mass spectrometry. Review of the patient medical records associated with the isolates indicated the potential for sexual transmission among MSM for 5 of the 9 isolates, while 3 of the 4 non-MSM isolates were associated with recent travel to Asia (Table 1). Because of the well-documented outbreaks of multidrug-resistant *Campylobacter* spp in Québec [12, 15, 16], 9 multidrug-resistant *C. coli* isolates were also obtained from the Laboratoire de santé publique du Québec in Canada from the same time period. Seven of these 9 isolates from Canada were also associated with MSM. All isolates were then phenotypically confirmed, AST was performed as above, and WGS was performed to examine mechanisms of antimicrobial resistance, virulence factors, and epidemiological relatedness.

**Table 1.** Epidemiological Information for the Isolates in This Study

Location and Strain	*Campylobacter* Species	Date ofCollection	Age, y	Sex	MSM	HIV	Travel/Origin	Resistance
Québec								
42478	*C. coli*	Dec 2017	24	M	Y	N	…	FQ, AZM, TET, GEN
43371	*C. coli*	Dec 2017	44	M	Y	Y	…	FQ, AZM, TET, GEN
48777	*C. coli*	Jan 2018	40	M	Y	Y	…	FQ, AZM, TET
76331	*C. coli*	May 2018	48	M	Y	N	…	FQ, AZM, TET
138449	*C. coli*	Jan 2015	62	M	Y	Y	…	FQ, AZM, TET
143854	*C. coli*	Sep 2015	29	M	Y	N	…	FQ, AZM, TET
143970	*C. coli*	Sep 2015	59	M	Y	Y	…	FQ, AZM, TET
148558	*C. coli*	Apr 2016	74	M	N	NA	…	FQ, AZM, TET
158403	*C. coli*	Apr 2017	25	M	NA	NA	…	FQ, AZM, TET
Washington State								
SP15-082	*C. coli*	Jul 2015	68	M	N	N	Malaysia	FQ, AZM, TET
SP16-070	*C. coli*	Jun 2016	21	M	N	N	Thailand	FQ, AZM, TET
SP17-196	*C. jejuni*	Dec 2017	72	M	N	N	Philippines	FQ, AZM, TET
HMC314	*C. jejuni*	Jan 2018	55	M	Y	N	…	FQ, AZM
SP18-054	*C. coli*	Feb 2018	25	M	Y	N	…	FQ, AZM, TET, GEN
SP18-090	*C. coli*	Feb 2018	27	M	Y	Y	…	FQ, AZM, TET, GEN
S871	*C. coli*	Mar 2018	34	M	Y	Y	…	FQ, AZM, TET
SP18-164	*C. jejuni*	Jun 2018	22	M	Y	N	…	FQ, AZM
SP18-232	*C. coli*	Oct 2018	59	F	N	N	…	FQ, AZM, TET
Pathogen Detection (NCBI)								
PNUSAC002907	*C. coli*	Sep 2017	30-39	…	…	…	Midwest US	…
PNUSAC000107	*C. coli*	May 2015	20-29	…	…	…	Midwest US	…
PNUSAC000108	*C. coli*	Jul 2015	30-39	…	…	…	Midwest US	…
PNUSAC000199	*C. coli*	Nov 2015	50-59	…	…	…	Midwest US	…
PNUSAC000219	*C. coli*	Nov 2015	50-59	…	…	…	Midwest US	…
PNUSAC006454	*C. coli*	…	…	…	…	…	…	…
PNUSAC007077	*C. coli*	…	…	…	…	…	…	…
PNUSAC008980	*C. coli*	…	…	…	…	…	…	…
PNUSAC007971	*C. jejuni*	…	…	…	…	…	…	…
NC05-27	*C. jejuni*	2005	…	…	…	…	…	…
PNUSAC000631	*C. jejuni*	Jun 2016	20-29	…	…	…	Southwest US	…
PNUSAC001707	*C. jejuni*	…	…	…	…	…	…	…
PNUSAC004578	*C. jejuni*	May 2018	30-39	…	…	…	Midwest US	…
PNUSAC005510	*C. jejuni*	Jul 2018	20-29	…	…	…	Midwest US	…
PNUSAC005955	*C. jejuni*	Aug 2018	20-29	…	…	…	Midwest US	…
PNUSAC006340	*C. jejuni*	…	…	…	…	…	…	…
PNUSAC006599	*C. jejuni*	Mar 2018	20-29	…	…	…	Southeast US	…
PNUSAC006863	*C. jejuni*	Jun 2018	30-39	…	…	…	Southeast US	…
PNUSAC007906	*C. jejuni*	…	…	…	…	…	…	…

Colored rows differentiate genomic clusters.

Abbreviations: AZM, azithromycin; F, female; FQ, fluoroquinolone; GEN, gentamicin; HIV, human immunodeficiency virus; M, male; MSM, men who have sex with men; N, no; NA, not available; NCBI, National Center for Biotechnology Information; TET, tetracycline; US, United States; Y, yes.

In addition to resistance to fluoroquinolones and macrolides, 2 *C. coli* isolates from Washington State and 2 from Québec were resistant to gentamicin (Table 2). All *C. coli* isolates were also resistant to tetracycline.

**Table 2.** Antimicrobial Resistance Patterns of the *Campylobacter coli* and *Campylobacter jejuni* Isolates

Isolate	Minimum Inhibitory Concentration, μg/mL										
	AMC^a^	CIP^b,c^	ERY^b,c^	FOS	GEN^d^	MER^a^	TET^b,c^	RIF	AZM^d,e^	CLI^d^	CHL^d^
42478	4 (S)	>32 (R)	>256 (R)	16 (–)	>256 (R)	0.064 (S)	>256 (R)	>32 (–)	>256 (R)	32 (R)	8 (S)
43371	2 (S)	>32 (R)	>256 (R)	16 (–)	>256 (R)	0.32 (S)	>256 (R)	>32 (–)	>256 (R)	32 (R)	8 (S)
48777	4 (S)	>32 (R)	128 (R)	64 (–)	0.25 (S)	0.125 (S)	>256 (R)	>32 (–)	32 (R)	256 (R)	32 (R)
76331	4 (S)	16 (R)	>256 (R)	32 (–)	0.5 (S)	0.032 (S)	>256 (R)	>32 (–)	>256 (R)	8 (R)	16 (S)
138449	4 (S)	>32 (R)	>256 (R)	32 (–)	0.25 (S)	0.5 (S)	>256 (R)	>32 (–)	>256 (R)	8 (R)	8 (S)
143854	4 (S)	>32 (R)	>256 (R)	32 (–)	0.125 (S)	0.5 (S)	>256 (R)	>32 (–)	>256 (R)	8 (R)	8 (S)
143970	8 (R)	>32 (R)	>256 (R)	64 (–)	0.125 (S)	0.5 (S)	>256 (R)	>32 (–)	>256 (R)	16 (R)	8 (S)
148558	4 (S)	>32 (R)	>256 (R)	32 (–)	0.125 (S)	0.125 (S)	>256 (R)	>32 (–)	>256 (R)	8 (R)	8 (S)
158403	8 (R)	>32 (R)	>256 (R)	16 (–)	0.5 (S)	0.5 (S)	>256 (R)	>32 (–)	>256 (R)	16 (R)	16 (S)
S871	16 (R)	>32 (R)	>256 (R)	64 (–)	0.25 (S)	1.0 (S)	>256 (R)	>32 (–)	…	…	…
SP15-082	8 (S)	>32 (R)	>256 (R)	>1024 (–)	0.25 (S)	0.5 (S)	>256 (R)	>32 (–)	>256 (R)	16 (R)	32 (R)
SP16-070	8 (S)	>32 (R)	>256 (R)	32 (–)	0.5 (S)	0.5 (S)	>256 (R)	>32 (–)	>256 (R)	32 (R)	16 (S)
SP18-054	4 (S)	>32 (R)	>256 (R)	16 (–)	>256 (R)	0.064 (S)	>256 (R)	>32 (–)	>256 (R)	64 (R)	8 (S)
SP18-090	2 (S)	>32 (R)	>256 (R)	16 (–)	>256 (R)	0.032 (S)	>256 (R)	>32 (–)	>256 (R)	64 (R)	16 (S)
SP18-232	4 (S)	>32 (R)	128 (R)	64 (–)	0.125 (S)	0.5 (S)	>256 (R)	>32 (–)	>256 (R)	4 (R)	4 (S)
18–164	4 (S)	>32 (R)	>256 (R)	32 (–)	0.25 (S)	0.008 (S)	0.5 (S)	>32 (–)	>256 (R)	128 (R)	4 (S)
HMC314	4 (S)	8 (R)	>256 (R)	32 (–)	0.25 (S)	0.016 (S)	0.25 (S)	>32 (–)	>256 (R)	16 (R)	4 (S)
SP17-196	4 (S)	>32 (R)	>256 (R)	32 (–)	0.25 (S)	0.5 (S)	>256 (R)	>32 (–)	32 (R)	256 (R)	256 (R)

Susceptibility interpretations are from the Clinical and Laboratory Standards Institute (CLSI) and/or the European Committee on Antimicrobial Susceptibility Testing (EUCAST). (–) Indicates no CLSI or EUCAST interpretation.

Abbreviations: AMC, amoxicillin–clavulanic acid; AZM, azithromycin; CHL, chloramphenicol; CIP, ciprofloxacin; CLI, clindamycin; ERY, erythromycin; FOS, fosfomycin; GEN, gentamicin; MER, meropenem; R, resistant; RIF, rifampin; S, susceptible; TET, tetracycline.

^a^EUCAST pharmacokinetic/pharmacodynamic (non–species related) breakpoints version 9.0.

^b^CLSI M45 3rd edition: 2016 breakpoints.

^c^EUCAST breakpoints version 9.0.

^d^EUCAST epidemiologic cutoff value (accessed 6 August 2019).

^e^EUCAST breakpoints version 9.0; note that ERY susceptibility can be used to determine AZM susceptibility.

### Two Clusters of *C. coli* From Seattle and Montréal With Near Identity

Multilocus sequencing typing using the combined *C. jejuni* and *C. coli* typing scheme for our 18 *Campylobacter* isolates yielded 12 different sequence types (STs) (Supplementary Table 1) [25]. Only 2 of the STs contained >1 isolate. Notably, we identified novel *glyA*, *pgm*, tkt, and *uncA* alleles from isolate HMC314. In addition, we identified 6 new *Campylobacter* STs from our isolates, which have been deposited in PubMLST [21].

**Figure 1.**
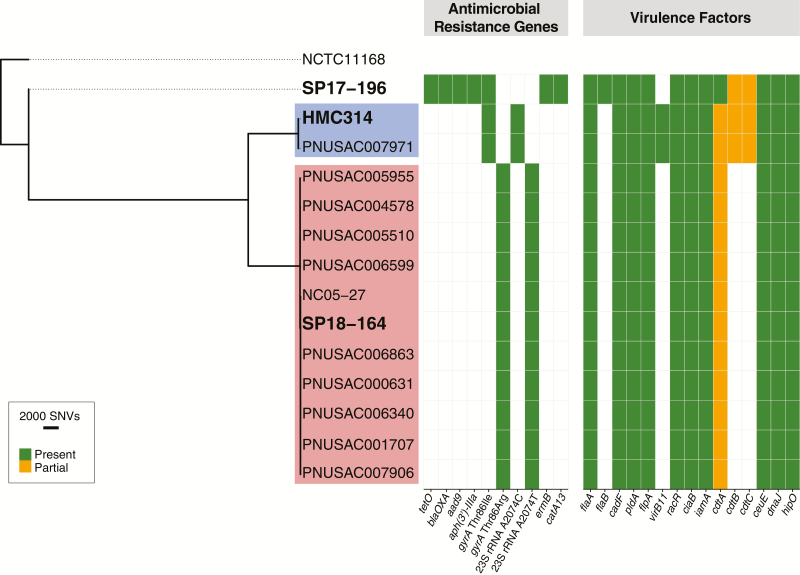
Core genome single-nucleotide polymorphism (SNP) phylogenetic tree for multidrug-resistant *Campylobacter jejuni* isolates. Isolates sequenced in this study (in bold) are shown along with closely related isolates identified by the National Center for Biotechnology Information’s Pathogen Detection database (individual SNP clusters are highlighted by color). Isolates in the blue cluster differed by 7 SNPs while isolates in the red cluster differed by 0–55 SNPs. Antimicrobial resistance genes and virulence factors are denoted for each isolate. Of note, the *cdtA-C* toxin locus was interrupted by frameshifts in every *C. jejuni* isolate in this study. Abbreviations: rRNA, ribosomal RNA; SNV, single nucleotide variant.

Next, we performed core genome SNP phylogenies to assess the genetic relatedness of the 3 *C. jejuni* and 15 *C. coli* isolates. No significant clustering was seen among the 3 *C. jejuni* isolates from Seattle, with 14 697–34 182 pairwise SNPs seen between each strain (Figure 1; Supplementary Table 2).

**Figure 2.**
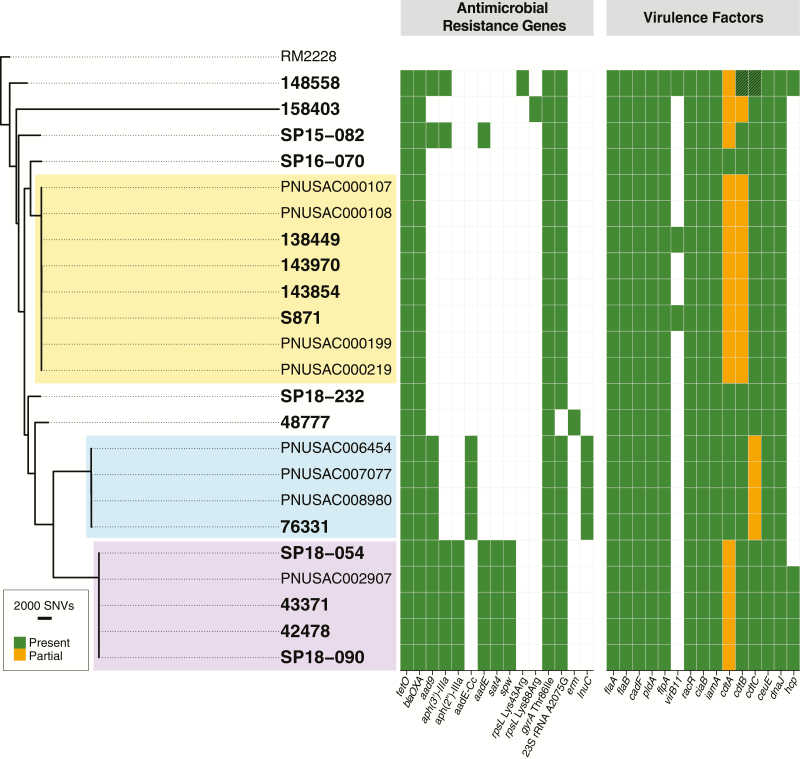
Core genome single-nucleotide polymorphism (SNP) phylogenetic tree for multidrug-resistant *Campylobacter coli* isolates. Isolates sequenced in this study (in bold) are shown along with closely related isolates identified by the National Center for Biotechnology Information’s (NCBI) Pathogen Detection database (individual SNP clusters are highlighted by color). By NCBI Pathogen Detection, the yellow cluster differed by 1–11 SNPs, the turquoise cluster differed by 3–10 SNPs, and the purple cluster differed by 1–7 SNPs. Antimicrobial resistance genes and virulence factors are denoted for each isolate. Of note, the *cdtA* gene was interrupted by frameshifts in almost every *C. jejuni* isolate in this study. The *cdtA-C* toxin locus was also interrupted by frameshifts in almost every *C. coli* isolate in this study. Abbreviations: rRNA, ribosomal RNA; SNV, single nucleotide variant.

The *C. coli* isolates differed from each other by an average of 11 132 pairwise core genome SNPs (range, 0–29 321). Two distinct clusters, each containing 4 *C. coli* isolates, were identified from our analysis. The first cluster contained 3 Montréal isolates and 1 Seattle isolate. The isolates in this cluster differed from each other by an average of 29 (range, 1–43) pairwise core genome SNPs (Figure 2; Supplementary Table 3). Of note, the Seattle isolate S871 and the Montréal isolate 138449 in this cluster differed by only 6 pairwise SNPs. The second cluster contained 2 Montréal isolates and 2 Seattle isolates. This cluster showed minimal genomic variability with an average of 2.5 (range, 0–5) pairwise SNPs separating the 4 isolates.

Interrogation of the NCBI Pathogen Detection database queried >31 000 *C. jejuni/C. coli* isolate genomes for relatedness and confirmed the Seattle–Montréal clusters identified above. We further identified 11 additional *C. jejuni* isolates and 8 additional *C. coli* isolates that met the NCBI Pathogen Detection definition of an SNP cluster (maximum 50 SNPs by whole genome MLST [wgMLST]) with our 18 sequenced isolates (Figures 1 and 2). Core genome analysis confirmed the tight evolutionary relationship of the clusters detected by wgMLST analysis (Supplementary Tables 2 and 3). Of note, each of the *Campylobacter* isolates sequenced from MSM in Washington State yielded additional cluster isolates, whereas isolates not associated with MSM did not yield any additional isolates by SNP cluster. Sample metadata were available for 11 of the 19 newly identified isolates and demonstrated that 9 of the isolates came from 20- to 39-year-olds, with 8 of them deriving from Midwestern states in the United States (US Department of Health and Human Services region 5; Table 1). This age distribution was significantly different from that of 5039 *Campylobacter* isolates in the NCBI Pathogen Detection database collected between 2014 and 2018 for which host age range information was available (Fisher exact test, *P* = .006; Supplementary Table 6). Antimicrobial resistance genes were identical within clusters, indicating that these additional isolates were also likely multidrug resistant, despite the lack of phenotypic resistance data.

### Antimicrobial Resistance Genes Readily Predict Antimicrobial Susceptibility Data

Consistent with a previous report, WGS analysis was highly accurate in predicting the ciprofloxacin resistance phenotype of the *Campylobacter* isolates [26]. In 17 of 18 isolates, the well-described fluoroquinolone resistance Thr86Ile *gyrA* mutation was identified (Figures 1 and 2; Supplementary Table 4) [27]. The less frequently encountered fluoroquinolone resistance–associated Thr86Arg *gyrA* mutation was identified in the remaining isolate, SP18-164 [28].

Tetracycline resistance was also accurately predicted from the sequencing analysis, as all 16 tetracycline-resistant isolates contained the *tetO* gene and the 2 tetracycline-susceptible isolates did not contain *tetO* [29]. In the single isolate with a significantly elevated MIC to chloramphenicol, we were able to identify the *cat* chloramphenicol resistant determinant, which was not present in the other 17 strains [30].

The 4 isolates displaying resistance to gentamicin each contained 5 aminoglycoside resistance determinants: *aad9*, *aadE*, *aph(3*’*)-IIIa*, *aph(2*″*)-IIIa*, and *spw* [31]. This combination of aminoglycoside resistance genes was unique to the gentamicin-resistant strains. Three of these determinants, *aad9*, *aadE*, and *aph(3*’*)-IIIa*, as well as the *aadE-Cc* determinant [31] and the aminoglycoside resistance–associated mutations in *rpsL* (Lys43Arg and Lys88Arg), were identified in 6 gentamicin-susceptible strains [32]. It is unclear if these genes and resistance-associated mutations confer resistance to other aminoglycosides.

We also identified the lincosamide resistance gene *lnuC* in 1 *Campylobacter* strain, the streptothricin resistance determinant *sat4* in 4 isolates, and genes encoding OXA-61 family β-lactamases in 16 isolates. However, the impact of these genes on phenotypic resistance is unclear.

We were further able to determine the genetic basis of erythromycin for all 18 *Campylobacter* isolates by screening for point mutations in the 23S ribosomal RNA (rRNA) and the acquired *erm* erythromycin resistance determinants. We identified 23S rRNA mutations (A2074G, A2074C, or A2075T) associated with macrolide resistance in 16 of the strains [33]. The 1 *C. jejuni* isolate without a macrolide resistance–associated mutation in 23S rRNA carried the resistance determinant *ermB*.

### Identification of a Novel *erm* Resistance Gene in the CRISPR Array

**Figure 3.**
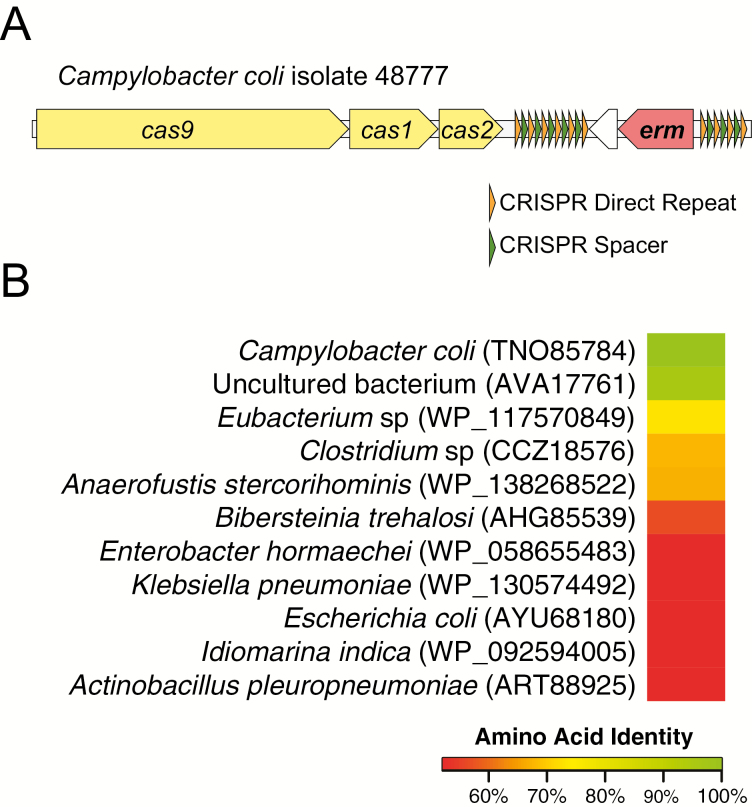
Novel erythromycin resistance gene detected in the clustered regularly interspaced short palindromic repeats (CRISPR) repeat array locus in *Campylobacter coli* isolate 48777. *A*, The *erm* gene was located downstream of the cas9-cas1-cas2 locus between 6 and 4 CRISPR direct repeats and a neighboring hypothetical protein. The translated amino acid sequence of this gene most closely aligned with an Erm protein from an uncultured bacterium (*B*). The next-highest hits by BLASTP were <75%, suggesting that these 2 *erm* genes form a novel erythromycin resistance gene family. Abbreviations: CRISPR, clustered regularly interspaced short palindromic repeats.

The remaining *C. coli* strain contained a novel *erm* gene (TNO85784.1) (Figure 3A). The encoded Erm protein was highly similar (96% identity by amino acid) to a 23S methyltransferase from an uncultured bacterium (AVA17761.1) that demonstrated high levels of resistance to erythromycin but not azithromycin when characterized in vitro [34] (Figure 3B). Whereas this Erm-containing isolate was resistant to both erythromycin and azithromycin, it had lower MICs to both of these drugs compared to other isolates. The next closest hit to the novel Erm protein by BLASTP shared 73% identity by amino acid to a 23S methyltransferase from a *Eubacterium* species (WP_117570849.1). No significant homology of the sequence could be found when querying the *Campylobacter* NCBI WGS database or BIGSI [35].

Analysis of locus surrounding the *erm* gene in isolate 48777 revealed that the Cas9-Cas1-Cas2 locus was located 770 bp downstream from the *erm* gene. Searching of this locus with the clustered regularly interspaced short palindromic repeats (CRISPR) CasFinder tool revealed 6 CRISPR direct repeats with 5 spacers downstream of the *erm* gene and 4 direct repeats and 3 spacers upstream of the *erm* gene [36] (Figure 3A). Sanger sequencing confirmed the genomic organization of the CRIPSR locus inferred from short-read sequencing. These data suggest that the novel *erm* may have been acquired by this *C. coli* isolate via recombination between repetitive sequences contained in a CRISPR array, making it the first such acquisition of *erm* in *Campylobacter* and the first detection of an antimicrobial resistance gene within a CRISPR array in any clinical isolate detected to date [37].

## DISCUSSION

The sexual transmission of enteric pathogens in MSM has long been recognized [8, 9, 12]. The emergence of multidrug resistance in *Shigella* spp associated with MSM is now known to result in large part from the global spread of specific clades [2, 3]. Here we show that multidrug-resistant lineages of *C. coli* are also spreading through sexual transmission in MSM across international boundaries. Outside of the MSM population, multidrug-resistant *Campylobacter* spp were also isolated from individuals in Seattle who recently returned from travel to Asia, suggesting 2 potential independent risk factors for acquisition of multidrug-resistant *Campylobacter*.

MSM are at increased risk for the acquisition of multidrug-resistant enteric pathogens from sexual practices resulting in fecal-oral transmission and from frequent exposure to antimicrobial agents for the treatment of sexually transmitted infections [38, 39]. The availability of preexposure prophylaxis to prevent HIV transmission may be promoting the spread of other sexually transmitted pathogens as a result of risk compensation [40].

Broad epidemiological data suggest that sexually transmitted *Campylobacter* infections in MSM may be underrecognized. Our data combined with increasing genomic surveillance of clinical *Campylobacter* isolates suggests that there are indeed clusters associated with transmission among MSM. We note that of the Washington State isolates, only *Campylobacter* sequenced from MSM yielded additional cluster isolates when interrogating NCBI databases. Although gender was not available for these additional isolates identified via genomic epidemiology, a skewed gender distribution of enteric infections has been suggested as a possible sign of enteric outbreaks among MSM in major metropolitan areas in the United Kingdom [41]. In addition to *Shigella*, a male skew for non-travel-associated enteric infections was found for *Campylobacter* [41]. Surveillance of MSM undergoing testing for rectal *Chlamydia* infections in the United Kingdom found that 1.8% of specimens contained *Campylobacter* spp, half of which were asymptomatic [42]. In the absence of specific treatment, asymptomatic carriage of enteric pathogens may persist for weeks after the clinical resolution of acute gastroenteritis, allowing ongoing transmission.

Conventional treatment of *Campylobacter* enteritis has relied on macrolides and fluoroquinolones. Due to increasing fluoroquinolone resistance, macrolides are considered the drugs of choice, but macrolide resistance due to 23S rRNA mutations is increasing, particularly in *C. coli* [33]. The optimal management of infections caused by macrolide- and fluoroquinolone-resistant strains is not established. Fosfomycin has been suggested as a possible alternative therapeutic agent [43].

The detection of a novel macrolide resistance determinant flanked by CRISPR direct repeats is intriguing. Although CRISPR-Cas systems are often viewed as a barrier to horizontal gene transfer, recent evidence suggests that they may also promote horizontal gene transfer [44]. Further investigation will be required to determine whether the association between *erm* and CRISPR-Cas in *C. coli* represents an unusual exception or a novel mechanism of antimicrobial resistance gene acquisition.

This study was chiefly limited by the small sample size and limited metadata associated with the isolates in the NCBI Pathogen Detection database. Nonetheless, it is remarkable that, in sampling cases from only 2 different cities, we found clonally related isolates to be associated with transmission among MSM. As isolates continue to be sequenced, we would hypothesize that the clusters identified here would grow in both number and scale.

The global emergence of multidrug-resistant enteric pathogens in MSM poses an urgent public health challenge that may require new approaches for surveillance and prevention. As *Campylobacter* isolates are not routinely submitted to the Washington State Public Health Laboratory, the relatedness of *C. coli* isolates from Seattle and Montréal was not detected from routine surveillance activities. Foodborne and sexually transmitted infections are traditionally the purview of separate branches of the public health hierarchy, but an integrated effort may be required to address this important problem. Rapid advances in the genomic epidemiology of enteric infections may provide the means to integrate these efforts.

## Supplementary Data

Supplementary materials are available at *Clinical Infectious Diseases* online. Consisting of data provided by the authors to benefit the reader, the posted materials are not copyedited and are the sole responsibility of the authors, so questions or comments should be addressed to the corresponding author.

ciz1060_suppl_Supplementary_Material

ciz1060_suppl_Supplementary_Tables

## Notes

***Financial support.*** This work was supported by departmental funds from the Department of Laboratory Medicine, University of Washington School of Medicine, Seattle.

***Potential conflicts of interest.*** F. C. F. has received grants, personal fees, and nonfinancial support from BioFire and Cepheid; personal fees from the Infectious Diseases Society of America; and grants and nonfinancial support from EliTech, outside the submitted work. A. L. G. has received personal fees from Abbott Molecular, outside the submitted work. All other authors report no potential conflicts of interest. All authors have submitted the ICMJE Form for Disclosure of Potential Conflicts of Interest. Conflicts that the editors consider relevant to the content of the manuscript have been disclosed.
