# Supplementary material for: International Spread of Multidrug-Resistant Campylobacter coli in Men Who Have Sex With Men in Washington State and Québec, 2015–2018
Source: Clin Infect Dis. 2019 Oct 29;71(8):1896–904. doi: 10.1093/cid/ciz1060 (PMC7643735; doi:10.1093/cid/ciz1060)
Supplement: ciz1060_suppl_Supplementary_Material [file ciz1060_suppl_supplementary_material.docx]

**Supplemental Material**

*Virulence factors*

To assess the pathogenicity of the *Campylobacter* strains, we screened the assemblies for 17 previously described virulence factors involved in motility, adhesion, invasion, toxin production, iron uptake and stress responses (Figures 1 and 2; Table S5). Nine of these factors – the motility factor *flaA*, four adhesion-related genes (*cadF*, *pldA*, *flpA*, *racR)*, the two invasion factors (*ciaB*, *iamA*), the iron uptake gene *ceuE*, and the stress response factor *dnaJ*, were present in all 18 isolates. The motility factor *flaB*, which is encoded directly adjacent to *flaA*, was present in all 16 *C. coli* and 1 *C. jejuni* isolates [37]. Interestingly, the 2 *C. jejuni* isolates lacking *flaB* appeared to encode 2 near-identical copies of *flaA* in tandem. All 3 of the *C. jejuni* isolates contained the hippuricase gene *hipO*. Of note, other *Campylobacter* species, including *C. coli*, do not encode hippuricases [38].

We identified all three genes of the cytolethal distending toxin locus, *cdtABC*, in 17 of the isolates. However, only 3 *C. coli* isolates had a fully intact *cdtABC* locus. In the other 12 *C. coli* and 2 *C. jejuni* isolates, at least one of the three proteins was prematurely truncated due to nonsense or frameshift mutations or recombination. Furthermore, in one of these isolates, genomic reorganization resulted in the separation of the *cdtABC* locus in the intergenic region between the *cdtB* and *cdtC*. Typically, the three genes of this locus are encoded in tandem [39]. However, in this isolate, *cdtA* and *cdtB* were encoded together, while *cdtC* was located 39.3 kb downstream of *cdtB*. The remaining *C. jejuni* isolate contained a truncated CdtA, but not CdtB or CdtC.

Four of the *C. coli* isolates contained the virulence factor *hcp*, which encodes the haemolysin of a type VI secretion system [40]. Notably, 3 of the *hcp*-positive isolates belonged to the same SNP cluster. The fourth isolate in this cluster (SP18-054) did not contain *hcp*. The type VI secretion system is encoded on a plasmid in *C. coli*. Thus, it is likely that isolate SP18-054 did not acquire the *hcp*-containing plasmid [41]. Compared to other *hcp*-positive isolates in the SNP cluster, SP18-054 lacked contigs totaling 68.5 kb that demonstrated high sequence similarity (>99%) with previously sequenced *C. jejuni* and *C. coli hcp*-containing plasmids including pMTVDSCj13-2 (CP017417.1) and pCOS502 (CP018901.1) [42].

One *C. jejuni* isolate and three *C. coli* isolates contained *virB11*, which encodes a protein of the type IV secretion system. This factor is encoded on the pVir plasmid in *C. jejuni* [43]. Accordingly, in the *C. jejuni* isolate, we identified *virB11* on a 39.9 kb circular contig that had a two-fold increase in coverage related to the rest of the genome. Furthermore, this contig shared a sequence similarity of >90% with the previously sequenced *C. coli* pOR12Vir plasmid (CP013734.1).
